# Appendix

**Published:** 1826-12

**Authors:** 


CIRCUMSTANCES have induced the Editor of the Medical and Physical Journal to address the following Letter to Sir Henry Halford, as President of the College of Physicians.

*Henrietta-street, Cavendish-square; Nov. 10th, 1826.*

SIR,—Some public discussion having taken place with regard to the line of conduct adopted by the late Censors of the College of Physicians, in the questions which have recently been agitated in the College of Surgeons, and the part which I have taken on this occasion having been represented as disrespectful towards that body, of which I am a Licentiate, I take the liberty of soliciting your attention to the following observations.

On a recent occasion, a circumstance occurred at a meeting of the Trustees of the Hunterian Museum, by which the feelings of the Licentiates were hurt, as (till it was explained) it appeared to place in a conspicuous point of view the preference given to the Fellows. That the impression produced by this occurrence was of the nature I have stated, is well known; and several letters were addressed to me upon the subject, as Editor of a Medical Journal. One of these, being more temperate than the others, I published, appending to it a note, in which I remarked that the letter proceeded upon the "assumption" that the Trustees had unlimited power to admit whom they pleased; adding, that I should like to hear what the Censors had to say in explanation. It is quite obvious that an opportunity was thus afforded to the Censors of conciliating the Licentiates, by explaining the circumstance which had given rise to the misunderstanding. A month, however, elapsed, but no communication was made; and it was not till the ensuing Number of the "Medical and Physical Journal" had been completed, and was on the eve of being struck off, that a letter was received,—not, indeed, from the Censors, but from one individual among them, who expressly stated it to be on his own part. This letter reflected on me as requiring what no "liberal and intelligent" person would have deemed necessary; but, as it seemed intended to explain the circumstance which had prevented the Licentiates from being, in this instance, placed on the same footing with the Fellows, I was induced to stop the press for its insertion; having stated in the preceding paragraph, that, if the Censors had not the power to grant what was desired, "no impartial person" would "blame them."

But, in the remarks alluded to, and which have given so much offence to the late Censors, I commented on the questionable policy of their interfering with the Hunterian Museum at all at such a time; because I regarded their co-operation with those Surgeons whose avowed object was the abrogation of the Charter of their College, as calculated to embroil the two corporate bodies, and therefore as injudicious; as contrary to all established etiquette in the profession, and therefore as in bad taste. And, I ask, in the event of public meetings being held by the Licentiates to overthrow the authority, and destroy the Charter, of that learned body over which you preside,—I ask whether, under such circumstances, it would not be regarded as a most ungracious act, were the Council, or any other officers of the College of Surgeons, to avail themselves of any influence their official situations might accidentally give them, and to volunteer their services on the side of the Licentiates?

But, so far was I from wishing to implicate the College generally in the dissapprobation bestowed upon the Censors, that I expressly stated my remarks to be applicable to the latter "alone:" adding my belief that their interference at such a juncture was condemned by the members of their own body. This belief, which I still entertain, was founded on the information of those on whom I could depend; and, for the honour of the College, I trust that the feeling is general.

It would obviously have been unfair to attribute an act of the Trustees of the Hunterian Museum, to the Censors of the College of Physicians, had not one of them thought proper to publish a full and circumstantial detail of what had passed at one of the meetings, thus claiming for himself and his colleagues the merit, and consequently entailing upon them the responsibility, of the part which they had taken in the discussions. This letter, as well as that signed "A Friend to the late Censors," appeared in the "Lancet;" a paper which few have had the boldness thus *openly* to countenance.

The writer of this anonymous letter asserts that my exculpation of the College, above mentioned, is a "severe imputation" upon that body, and contrary to my obligation to do every thing "*in honorem Collegii;*"—that is, it is held up as a violation of my oath that I should presume to say that the Censors have acted injudiciously, or that any of their brethren think so. But, in order to make his case the stronger, he has given a quotation, with inverted commas, as my words; yet he has felt himself at liberty to alter and to strengthen the expression, converting the postulate "*we believe* to be condemned" into the simple asseveration "*is* condemned;" which, however, I regard as of no importance, farther than as it is an illustration of the spirit which pervades the whole.*

With a view to show that their conduct on this occasion was not disapproved of by the members of the College of Physicians, the "Friend to the late Censors" quotes the opinions of two individuals,—yours, Sir, and that of the senior Censor. With regard to you, we are informed that "a statement of their conduct" was deemed "perfectly satisfactory;" but, as no part of the statement is given, and of your answer, "written by the Registrar," only the two words above quoted, it is obviously impossible to know of what nature the statement was, or how far the approval extended. One thing, however, is quite clear, that a "statement" *was* deemed necessary; a circumstance at variance with the assertion of this writer, that he has heard "of no one besides Dr. Macleod who condemns them:" for I have not the vanity to suppose that my opinion would have been regarded as sufficiently important to induce the Censors of the Royal College of Physicians to draw up a "statement of their conduct."

If you, Sir, really deemed their interference with the affairs of another corporate body, at a time when they were assailed by a disaffected party among themselves, "perfectly satisfactory," I can only regret that the opinion of so eminent an individual should in this respect be opposed to that of a large proportion of the respectable part of the profession. But if it should appear (as I have *strong* reason to believe) that Dr. Macmichael did not write *as Registrar*, and that the statement, together with the approval, had reference solely to the part which the Censors had taken with regard to the Licentiates, and to the circumstance of a Treasury minute putting it out of the power of the Trustees to admit them on the same terms as the Fellows, then it will remain for the "Friend to the late Censors" to explain on what grounds he has taken upon himself to extend your satisfaction touching one particular point into a general approval of their conduct with respect to the College of Surgeons.

But (to complete the absurdity,†) the attendance of the Censors at the Board of Trustees of the Hunterian Museum is represented by their "Friend" as a point of conscience, touching which they "swore a solemn oath," and "publicly received the Sacrament." Now, Sir, if it really was so imperative a duty, and if they have done all this for conscience sake, I cannot but think it a "severe imputation" upon you and Dr. Frampton, higher and senior officers, as well as men of more influence, to have absented yourselves from every one of these meetings, and left to the juniors the undivided honours of the whole transaction.

In the letter alluded to, it is acknowledged that the Censors were advised to send their explanation to the Medical and Physical Journal. Notwithstanding this recommendation, which sufficiently shewed the channel which *you* regarded as most proper for its communication, the "Friend to the late Censors" selected the "Lancet!" a journal teeming with abuse of the College and its members; in which, accordingly, a document, but not the "statement" appeared, the week after an illiberal attack upon that body, which I had absolutely refused to publish.

I fearlessly refer to the pages of the "London Medical and Physical Journal," as an index of my feelings towards the College of Physicians, and as the best proof that I have never endeavoured to create or to foster dissentions among men, whose mutual interest it is to avoid such unpleasant discussions. I beg, however, not to be misunderstood. I do not wish to exculpate myself from the charge of having censured the conduct of the late Censors, which, as a public journalist, and touching a public question, I had a right to do; but I do wish to rebut the accusation of having thereby deviated from the consistent line of conduct which I have throughout observed towards the College of Physicians as a body, and towards the profession in general.

* This writer calls upon me to give him the names of those who condemn the conduct of the late Censors. Had I accused them, on the authority of others, of any immoral act, he would have had a right to make this demand; but, as the extent of my censure implied only a doubt of their absolute wisdom, it is too much to expect that I should obey such an unreasonable request. For me to do so would be to take dishonourable advantage of opinions expressed in the course of conversation: at the same time I am satisfied, from the character of the parties, that, were it of any importance for the Censors to be acquainted with their names, they would not be deterred from declaring themselves by the vapouring threat about exclusion from College offices, or from any dread of being regarded by this writer as "deficient in sense of moral obligation."

† These words were unfortunately omitted in the copies of this letter originally struck off; which, we fear, may have rendered the meaning less obvious.

I have addressed this letter to you, Sir, in the present form, because I have too high a respect for you, and too much regard for my own character, to think of giving it publicity through the same medium which the "Friend to the late Censors" has had the indiscretion to adopt: and I must say that I should, indeed, regard myself as deserving the "strongest reprobation," had I presumed to offer such an insult to the College of Physicians as I conceive him to have done, by selecting, as the channel of his communication, a paper—of which it would not become me to speak in the terms which it merits. Indeed, when I consider the medium through which the letters in question have been published, I feel that some apology is necessary for noticing them at all; and I certainly should not have done so had not the circumstances under which they appeared given to them somewhat of an official form; the latter containing references to various papers, which has led to the belief that it proceeds from the late Censors themselves.

I trust that, in the foregoing remarks upon the conduct of the late Censors, which the circumstances have rendered unavoidable, I have not departed from that temperance of language and fair line of argument which Gentlemen ought to adopt towards each other: and in conclusion I beg to say, that I shall not be induced, by any consideration whatever, to prolong this discussion. It would, indeed, be endless, were the Editor of a Journal liable to be drawn into a controversy with every one from whom he differed in opinion. Both sides of the question have now been stated, and those who feel any interest about it will form their own judgment on the subject.

I have the honour to be, Sir, your most obedient humble servant,

RODERICK MACLEOD,
Licentiate of the Royal College of Physicians, and
Editor of the "London Medical and Physical Journal."

*To* SIR HENRY HALFORD, BART.
President of the Royal College of Physicians.

P.S. *(Saturday, Nov. 11th.)*—I have just been informed that the anonymous letter, so frequently alluded to, has been acknowledged, in the Lancet of to-day, as having proceeded from one of the late Censors.—I take this opportunity of stating that it is not my intention to read, and consequently that I cannot answer, any thing which may hereafter appear in that publication.

---

At the same time that the above Letter was in circulation, an Address, of which the following is a copy, appeared from Dr. AGER.

(CIRCULAR.)

The London Medical and Physical Journal for September last contained a letter from an anonymous Licentiate, complaining, that the right of claiming admission, and of introducing visiters to the Hunterian Museum, was restricted to the Fellows of the College of Physicians and the Members of the College of Surgeons; and proposing, that a remonstrance should be sent by the Licentiates to the *Trustees*, on the supposition, that they had made the regulation. The Editor of the Journal, Dr. Macleod, observed, "if this be correct, there can be but one opinion of the transaction;" and then demanded an explanation from the *Censors*. Having accidentally seen these remarks, I considered it my duty to point out the error at once to the President in a letter, dated on the 13th of the same month. It was thought best to enable the Editor to correct the mistake through the medium of Dr. Chambers: who, on the 26th, wrote that "Macleod wished to be entirely impartial on the subject of the Censors, and would have been very glad of any good answer to the Licentiate's letter;" and requested the Registrar, "if there appears to Sir Henry no objection to the publication of Dr. Ager's Remarks," to send them in the course of the day for that purpose. This was communicated to me, at the President's desire, on the following morning, with a request, that I would send an explanation to the Editor as soon as possible, lest the reputation of the College should suffer from continual misrepresentations remaining uncontradicted. I therefore wrote immediately to Dr. Macleod, observing, that such explanation from the Censors appeared to me "hardly necessary; for surely every liberal and intelligent person would conclude, that the regulation did not proceed from them: however, I can have no objection to its being stated on my part, that it was one of the original conditions, comprised in a Treasury-Minute, when the Museum was presented to the College of Surgeons, which the Trustees were merely appointed to see enforced." This letter was published in the Journal for October, preceded on the same page by farther strictures on the conduct of the Censors, asserting a belief, that their "officious interference with the College of Surgeons," is "condemned by none more heartily than by the members of their own body;" calling in question "their taste and judgment in interfering in the business at all;" and yet insisting, that if they could have procured for the Licentiates the right of admission, as they have not done so, "nothing that has been said of them is too severe, and nothing that we have said is severe enough."* Having drawn up the following statement of facts, in the accuracy of which the two junior Censors concurred, I thought it proper to show it to Dr. Frampton, as the charge unjustly implicated him also. He at once declared, that he entirely approved of what had been done; and candidly added, that if he had not, it was his duty to have attended the meetings, and opposed it. He also agreed with us in the propriety of submitting the whole matter to the President, since an imputation was thrown upon the College rather than ourselves; and leaving it to his discretion to take any steps which he might think the interests of that body required on the occasion. This was accordingly done; and the President has replied through the medium of the Registrar, that "he thinks the statement perfectly satisfactory," and recommended its being made public. I have felt some difficulty about the best method to be adopted for that purpose. Of course, the London Medical and Physical Journal was entirely out of the question: but thinking it particularly important, that these transactions should be fully known to the Members of the College, I have determined to distribute a circular letter among them, and a few other friends. (Signed) JOSEPH AGER.

STATEMENT OF FACTS.

When the Hunterian Museum was presented to the late Corporation of Surgeons, a Treasury-Minute of the Conditions was drawn up, and certain Trustees were appointed to see them enforced; among whom are the President and four Censors of the College of Physicians. The first condition is, that "the Collection shall be open four hours in the forenoon, two days every week, for inspection and consultation of the Fellows of the College of Physicians, the Members of the Company of Surgeons, and persons properly introduced by them; a Catalogue of the preparations, and a proper person to explain it, being at those times always in the room." The number of days of admission had been diminished, to give more time for proceeding with the Catalogue; and a general complaint was made among the Members of the College of Surgeons, that the Museum was not sufficiently open and useful to them.

At the Quarterly Meeting of the Trustees on the 6th of May last, there were present, the Duke of Somerset, Mr. Davies Gilbert, Dr. Ager, Dr. Elliotson, and Dr. Ramadge. The last gentleman suggested to the Board the propriety of making the days of admission more frequent, in which all the other Trustees concurred: but it was thought advisable, that an extraordinary meeting should be called, and the Curators summoned to attend on the 20th of May, to enable the Board to decide finally upon that subject, as well as the means of completing the Catalogue as soon as possible.

At this second meeting, besides the Trustees present at the preceding, Lord Colchester and Sir Everard Home attended; but the latter gentleman withdrew on the introduction of the Curators. Arrangements were made for expediting the preparation of the Catalogue, and it was determined unanimously, that the original conditions should be strictly enforced.

At the next Quarterly Meeting, on the 5th of August, there assembled Lord St. Helens, Sir E. Home, Dr. Ager, and Dr. Ramadge. A communication was received from a Meeting of Members of the College of Surgeons, conveying their thanks for what had been already done; and requesting, that the times of admission to the Museum should be increased, and the Licentiates of the College of Physicians, as well as other respectable Medical and Scientific persons, privileged to attend. These regulations had indeed been suggested to the Board by the Censors present at the former Meetings; but it was seen with regret, on referring to the original conditions, that the Trustees had no power to enforce them. The only arrangement then made respected the interpretation of the terms "properly introduced." A personal introduction had previously been required; but it was unanimously agreed, that a letter should be thenceforth considered sufficient, and that the regulation thus interpreted should be communicated to those Gentlemen who had applied to the Board.

---

This address does not appear to us to require any answer farther than may be found in our Letter. The only circumstance to which we shall allude is this: Dr. Ager has introduced Dr. Chambers's name in such a manner as seems to imply an idea that he was concerned in the paragraph which has given offence to the late Censors: we think it right to state unequivocally, that he had nothing whatever to do with it, and did not even know of it till it was published; neither, we may add, is he at all aware of this declaration on our part.

---

* *We request our readers to compare the above with the original passage to which it refers, No. for Oct. pages* 390-1.

